# Determinants of the low use of Thailand’s Universal Coverage Scheme: a national cross-sectional study

**DOI:** 10.3389/fpubh.2024.1475319

**Published:** 2024-11-22

**Authors:** Seung Chun Paek, Ning Jackie Zhang

**Affiliations:** ^1^Department of Society and Health, Faculty of Social Sciences and Humanities, Mahidol University, Salaya, Thailand; ^2^Health Solution Research Unit, Faculty of Social Sciences and Humanities, Mahidol University, Salaya, Thailand; ^3^College of Health Professions, Grand Valley State University, Grand Rapids, MI, United States

**Keywords:** Universal Coverage Scheme, 30-baht health insurance, public health insurance, healthcare use, healthcare access, Thailand

## Abstract

**Introduction:**

Thailand’s Universal Coverage Scheme (UCS) has increased overall healthcare use by offering free healthcare for about 76% of the entire population since it was introduced in 2002. However, a considerable number of beneficiaries have continued to depend on private healthcare, and the low use of the UCS has been cited as a challenge to accomplishing the policy’s goal of universal access to healthcare. Thus, this study divided healthcare use into three patterns (self-medication, private providers, and UCS) and investigated the socio-demographic characteristics of non-users of the UCS and their reasons for non-use.

**Methods:**

A cross-sectional quantitative analysis was performed using data from the 2019 Health and Welfare Survey. UCS beneficiaries aged 15 years or older who had used healthcare during the past month were included in the sample. Descriptive analysis and multinomial logistic regression were performed to analyze associations between patterns of healthcare use and socio-demographic factors chosen based on Aday and Andersen’s access to medical care model.

**Results:**

Of the study sample (*n* = 5,636), about 46.1% used healthcare services outside the UCS delivery system, of whom 33.8 and 12.3% used self-medication and private healthcare providers, respectively. Non-users generally had a higher socio-demographic status than UCS users. Specifically, they were young, had a high income, were employed, lived in urban areas, or did not have a chronic disease. The most common reason for non-use of the UCS was accessibility barriers (59.6%; e.g., long queues in public providers), followed by availability (25.4%; e.g., limited operating hours of public providers) and quality barriers (14%; e.g., unsureness of the quality of medicine offered by public providers). Moreover, self-medication users tended to be concerned about availability barriers, while private-provider users tended to be concerned about quality barriers for using the UCS.

**Conclusion:**

Under the UCS policy, there is a gap between the demands for healthcare and the resources assigned to increase the capability of public healthcare providers. That is, the UCS has increased financial accessibility for the use of the UCS (i.e., free healthcare from public providers). However, it probably has not yet increased healthcare resources and infrastructure facilitating the use of the UCS. This may have prevented the UCS from meeting the demands of its intended beneficiaries, especially those in high socio-economic groups, and ultimately forced them to use private healthcare.

## 1 Introduction

Thailand offers universal healthcare coverage by implementing three public health insurance policies: the Civil Servant Medical Benefits Scheme (CSMBS), the Social Security Scheme (SSS), and the Universal Coverage Scheme (UCS). The CSMBS, SSS, and UCS are insurance policies for people in the public, private, and informal employment sectors, and they cover about 10, 14, and 76% of the population, respectively (1, 2). The implementation costs of all three schemes, including managerial costs of the system of public healthcare providers, accounted for about 70.4% of the nation’s total health expenditure in 2021 (3).

The UCS, which is the largest insurance policy, was introduced in 2002 and has since offered free healthcare to about 76% of the population in the informal employment sector (2). The UCS is a tax-financed non-contributory policy. The policy, also known as “30-baht health insurance,” offers comprehensive healthcare benefits for 30 Thai baht, which is equal to about 0.90 US dollars. The benefits include acute and rehabilitative healthcare and preventive healthcare efforts, such as routine health checks and essential immunizations. The policy requires beneficiaries to receive healthcare at designated healthcare providers, namely public primary healthcare providers. Beneficiaries who bypass their designated providers have to pay for healthcare out of pocket (4, 5).

The effects of the UCS on healthcare use in Thailand have been extensively studied (2, 6–11). The overall pattern indicates that the UCS has increased overall healthcare use, especially among low socio-demographic groups. Specifically, trend analyses have revealed that the use of public healthcare providers (e.g., hospitalizations and outpatient visits) increased persistently after the UCS was implemented (2, 6, 7). Moreover, studies investigating patterns of healthcare use have revealed that the UCS has increased the use of public healthcare providers while decreasing the use of private modes of healthcare, such as self-medication (e.g., over-the-counter or Thai traditional medicines) and private healthcare providers (e.g., private clinics or hospitals). This pattern was more evident in low socio-demographic groups than in high socio-demographic groups (8–11).

Interestingly, although the UCS has increased overall healthcare use by offering free healthcare, a significant number of beneficiaries have continued to use private healthcare instead of the UCS (11–13). Specifically, a study using national survey data from 2004 showed that about 50% of UCS beneficiaries using healthcare sought care through private means, with about 32 and 18% using self-medication and private healthcare providers, respectively. These non-users generally had a higher socio-demographic status than users of the UCS. Specifically, they had a high income, were highly educated and employed, or lived in urban areas (11). The 50% non-use rate and associated socio-demographic patterns were also reported in two recent studies using the same data from 2007 (12) and 2013 (13), though the use rates of self-medication and private healthcare providers varied slightly across these studies.

Moreover, the low use of public health insurance has been reported in studies from other Southeast Asian countries, particularly Indonesia, Philippines, and Vietnam (12, 14–19). Consistent with previous results from Thailand, these studies indicate that the use of public health insurance increased while the use of private healthcare decreased after the policy implementation. However, the overall use of public health insurance was relatively low due to the high use of private healthcare and the high occurrence of bypassing in high socio-demographic groups. Specifically, a study conducted in Indonesia showed that among all beneficiaries of the National Health Insurance who had used healthcare over the past month, only 42% used the insurance. The other 58% used private healthcare, of whom about 53 and 5% used self-medication and private healthcare providers, respectively. These non-users had a high income, were highly educated and employed, or lived in urban areas (19).

Two contrasting opinions may exist regarding the low use of public health insurance (or the high use of private healthcare). On the one hand, the high use of private healthcare can be considered reasonable since individuals can freely choose the type of healthcare they receive according to their preferences and socio-demographic circumstances. As previous studies have revealed, individuals with a high socio-demographic status prefer private healthcare to public health insurance. If private healthcare is used by high socio-demographic groups because of relatively affordable costs and convenient accessibility, their high use of private healthcare is reasonable.

On the other hand, the high use of private healthcare could be partly due to issues with healthcare offered by public health insurance, such as low accessibility (e.g., long queues) and low availability (e.g., limited healthcare benefits). Such issues may give individuals no choice but to use private healthcare, even if they would rather use public health insurance. This perspective suggests that the high use of private healthcare indicates a gap between people’s needs and the provision of healthcare under a public health insurance policy, which should be reduced if the policy’s goal is to provide universal access to healthcare.

The stated objective of the UCS is “to equally entitle all Thai citizens to quality health care according to their needs, regardless of their socioeconomic status” [(2), p. 37]. That is, the UCS is an insurance policy founded on the principle of universal access to healthcare for all population groups, rather than particular groups such as low-income groups. Thus, the low use of the UCS is a challenge to accomplishing the policy objective of universal access to healthcare, which has also been cited in previous studies (12, 13).

Previous studies provided a comprehensive understanding of the non-use of the UCS and its associated socio-demographic factors. However, to our knowledge, specific reasons for the non-use of the UCS have been relatively understudied. In addition, since previous studies used relatively outdated data, a study using recent data is required to verify the reliability and validity of previous results. Therefore, the present study investigated patterns of healthcare use to identify determinants of the low use of the UCS. Specifically, by dividing healthcare use into three patterns (self-medication, private providers, and UCS), this study explored the socio-demographic characteristics of non-users of the UCS and their reasons for non-use.

## 2 Methods

### 2.1 Data and sample

The present cross-sectional quantitative analysis utilized data from the 2019 Health and Welfare Survey (HWS). The HWS is a national survey providing comprehensive socio-demographic and healthcare-related parameters about the population of Thailand. The survey consists of four main components: (a) socio-demographic characteristics, (b) diseases and healthcare use, (c) lifestyle risk factors, and (d) household characteristics. The National Statistical Office of Thailand conducts and administers the HWS biannually.

The purpose of the HWS is to generate reliable representative statistics at the national level. For this purpose, a stratified two-stage sampling approach is used. Specifically, the survey’s scope encompasses households in all municipalities (i.e., urban areas) and non-municipalities (i.e., rural areas) in all 77 provinces of Thailand. The first stratum includes all 77 provinces and the second stratum includes two substrata (i.e., all municipalities and non-municipalities) in each province.

In stage 1, enumeration areas from all municipalities and non-municipalities are chosen according to proportional probability to the population’s size. In stage 2, 12–16 households from the chosen enumeration areas in municipalities and 8–12 households from those in non-municipalities are randomly chosen. Accordingly, the HWS can represent a national cross-section of all 77 provinces of Thailand, with equal-sized samples from each province (20).

The 2019 HWS was used because it is the latest report that provides all variables of interest in this study. The National Statistical Office of Thailand carried out the HWS in 2021 and 2023, but the datasets are not suitable for this study. The 2021 HWS was conducted during the COVID-19 pandemic and was not publicly released, and the 2023 HWS does not offer several key pieces of socio-demographic information, including income.

UCS beneficiaries aged 15 years or older who had used healthcare in the past month comprised the sample for analysis. Specifically, the 2019 HWS originally included a total of 63,594 respondents (23,549 households). Among the 63,594 respondents, 52,921 (about 83%) were aged 15 or above. Among them, 40,263 (about 76%) were UCS beneficiaries. Among the beneficiaries, 5,636 (about 14%) had used healthcare in the past month and they were ultimately used as the study sample for analysis.

### 2.2 Variables and measurement

The pattern of healthcare use (the outcome variable) was measured as a nominal variable with three categories: self-medication, private providers, and UCS. Among the study sample, those using over-the-counter or Thai traditional medicines were included in the category of self-medication, those using private clinics or hospitals were included in the private providers category, and those using the UCS were included in the category of UCS.

Independent variables were chosen based on Aday and Andersen’s behavior model of access to medical care (21, 22). This model divides factors associated with healthcare use into three components: predisposing, enabling, and need-for-care factors. Predisposing factors are individual demographic and socio-cultural characteristics determined before the incidence of an illness. Enabling factors refer to individual and community resources facilitating healthcare access. Need-for-care factors refer to subjective (or illnesses) and objective health problems (or diseases) generating the demand for healthcare. The present study used two demographic characteristics (age and sex), five socio-economic status indicators (income, education, employment, marital status, and place of residence), and the presence of chronic disease as predisposing, enabling, and need-for-care factors in the analysis, respectively.

Regarding measurement, age was a nominal variable with four categories (15–30, 31–45, 46–60, and 61 years or older). Income was measured as an income quartile (Q1–Q4) based on household equivalence scale income (23). Marital status (single, married, and divorced) and education (low, middle, and high) were used as a nominal variable and an ordinal variable with three categories, respectively. Regarding marital status, the “divorced” category included individuals who were divorced, widowed, or separated. Regarding education, the “low” category referred to primary school or lower, the “middle” category referred to secondary school, and the “high” category referred to college or higher. The rest of the variables—namely, sex (male and female), employment (yes and no), place of residence (urban and rural), and chronic disease (yes and no)—were dichotomous variables.

In addition, reasons for the non-use of the UCS were classified into four components (availability, accessibility, acceptability, and quality) based on the World Health Organization’s Availability, Accessibility, Acceptability, and Quality (AAAQ) framework (24, 25). Barriers related to availability refer to the adequacy of the amount and type of healthcare (e.g., limited healthcare benefits). Barriers related to accessibility indicate the physical (e.g., long distances or long queues) and financial (e.g., costs of healthcare) accessibility of healthcare. Acceptability barriers are related to inequality and unfairness in the provision of healthcare (e.g., patient discrimination or unfriendly staff). Finally, quality barriers refer to acceptable healthcare standards (e.g., perceived quality of healthcare). This study explored reasons for not using the UCS according to these four components and the classified patterns of healthcare use.

### 2.3 Statistical analysis

Descriptive statistical analysis was first performed to offer summary statistics of the study sample and variables. The bivariate association between the outcome variable (i.e., patterns of healthcare use) and each of the independent variables was then investigated by performing a chi-square test. Then, because the outcome variable was a nominal variable with three categories (self-medication, private providers, and UCS), a multinomial logistic regression (MLR) model was established to investigate the outcome’s multivariate associations with the independent variables (26, 27).

The performance of the MLR model was evaluated by the Pearson’s goodness-of-fit test. In addition, this study inspected multicollinearity across the independent variables by using the variance inflation factor and the cross-comparison between the crude odds ratio (COR) and adjusted odds ratio (AOR). The variance inflation factor scores ranged from 1.037 to 3.194. Moreover, considerable directional changes between the COR and AOR were not detected in the cross-comparison, indicating the absence of multicollinearity in the MLR model (27). The entire results of the MLR model are presented in Appendix.

Statistical significance was assessed at a *p*-value of 0.05. For the MLR model, this study used the OR and 95% confidence interval (CI) to assess the directional relationship and statistical significance. IBM SPSS Statistics version 20 was used for all statistical analyses.

## 3 Results

### 3.1 Descriptive statistical analysis

Table 1 reveals the results of the descriptive statistical analysis. Among 5,636 UCS beneficiaries aged 15 years or older who had used healthcare over the past month, 53.9% (*n* = 3,038) used the UCS. The other 46.1% (*n* = 2,598) used private healthcare, with 33.8% (*n* = 1,904) and 12.3% (*n* = 694) using self-medication and private healthcare providers, respectively. Non-users (both self-medication and private-provider users) generally had a higher socio-demographic status than UCS users.

**Table 1:** Results of descriptive statistical analysis.

Variables			Pattern of healthcare use						*X*^2^ test *p*-value
	Overall (*n* = 5,636; 100%)		UCS (*n* = 3,038; 53.9%)		Self-medication (*n* = 1,904; 33.8%)		Private provider (*n* = 694; 12.3%)		
	*n*	%	*n*	%	*n*	%	*n*	%	
Predisposing factors									
Age									<0.001
15–30	461	8.2	153	33.2	228	49.5	80	17.4	
31–45	800	14.2	295	36.9	367	45.9	138	17.3	
46–60	1,772	31.4	887	50.1	666	37.6	219	12.4	
61 or older	2,603	46.2	1,703	65.4	643	24.7	257	9.9	
Sex									0.074
Male	2,222	39.4	1,160	52.2	789	35.5	273	12.3	
Female	3,414	60.6	1,878	55.0	1,115	32.7	421	12.3	
Enabling factors									
Income									<0.001
Q1	2,092	37.1	1,329	63.5	547	26.2	216	10.3	
Q2	1,512	26.8	789	52.2	556	36.8	167	11.0	
Q3	1,246	22.1	605	48.6	477	38.3	164	13.2	
Q4	786	14.0	315	40.1	324	41.2	147	18.7	
Education									<0.001
Low	4,306	76.4	2,506	58.2	1,338	31.1	462	10.7	
Middle	1,154	20.5	466	40.4	498	43.2	190	16.5	
High	176	3.1	66	37.5	68	38.6	42	23.9	
Marital Status									<0.001
Single	648	11.5	286	44.1	278	42.9	84	13.0	
Married	3,506	62.2	1,818	51.9	1,209	34.5	479	13.7	
Divorced	1,482	26.3	934	63.0	417	28.1	131	8.8	
Employment									<0.001
Yes	3,159	56.1	1,436	45.5	1,276	40.4	447	14.2	
No	2,477	44.0	1,602	64.7	628	25.4	247	10.0	
Place of residence									0.024
Urban	2,852	50.6	1,487	52.1	1,006	35.3	359	12.6	
Rural	2,784	49.4	1,551	55.7	898	32.3	335	12.0	
Need-for-care factor									
Chronic disease									<0.001
Yes	2,946	52.3	2,183	74.1	469	15.9	294	10.0	
No	2,690	47.7	855	31.8	1,435	53.4	400	14.9	

Regarding the predisposing factor, the use rate of private healthcare increased with age, indicating that young people tend to use private healthcare while old people tend to use the UCS. Specifically, the use rates of self-medication were 49.5, 45.9, 37.6, and 24.7% in the age groups of 15–30, 31–45, 46–60, and 61 years or older, respectively. For the private providers group, the use rates were about 17.4, 1.73, 12.4, and 9.9% for the same respective age categories. Meanwhile, the use rates of the UCS were about 33.2, 36.9, 50.1, and 65.4% in the same respective categories. However, patterns of healthcare use were not different between the sexes.

In terms of the enabling factors, high socio-economic groups tended to use private healthcare while low socio-economic groups tended to use the UCS. Specifically, the use rates of self-medication (e.g., Q4: 41.2% vs. Q1: 26.2%) and private providers (e.g., Q4: 18.7% vs. Q1: 10.3%) in people with high incomes were higher than in those with low incomes. Highly educated people had higher use rates of self-medication (e.g., Q1: 31.1% vs. Q4: 38.6%) and private providers (e.g., Q1: 10.7% vs. Q4: 23.9%) than people with a low education level. Employed people had higher use rates of self-medication (yes: 40.4% vs. no: 25.4%) and private providers (yes: 14.2% vs. no: 10.0%) than unemployed people. People who were married and single, compared to those who were divorced, had higher use rates of self-medication (e.g., married: 34.5% vs. divorced: 28.1%) and private providers (e.g., married: 13.7% vs. divorced: 8.8%). Regarding place of residence, the use rates of self-medication (urban: 35.3% vs. rural: 32.3%) and private providers (urban: 12.6% vs. rural: 12.0%) were higher in urban people than in rural people.

Regarding the need-for-care factor, patients without a chronic disease tended to use private healthcare, while patients with a chronic disease tended to use the UCS. The use rates of self-medication (yes: 15.9% vs. no: 53.4%) and private providers (yes: 10.0% vs. no: 14.9%) were higher in people without a chronic disease than in those with a chronic disease.

In summary, about 46.1% of all UCS beneficiaries using healthcare services used them outside the UCS delivery system, of whom 33.8 and 12.3% used self-medication and private healthcare providers, respectively. Compared to UCS users, non-users generally had a higher socio-demographic status. They were younger, had a higher income, were highly educated, employed, married, living in urban areas, and did not have a chronic disease.

### 3.2 Multinomial logistic regression analysis

Table 2 presents the results of the MLR model. The *p*-value of the Pearson chi-square goodness-of-fit test was 0.739, indicating that the MLR model did not reveal a lack of fit to the data. Consistent with the descriptive statistical analysis, the MLR model reveals that non-users of the UCS were more likely than users to have a high socio-demographic status.

**Table 2:** Results of multinomial logistic regression analysis.

Variables	Self-medication (vs. UCS)				Private provider (vs. UCS)			
	AOR	95% CI	*p*-value	VIF	AOR	95% CI	*p*-value	VIF
Predisposing factor								
Age								
15–30	1.53	(1.11, 2.11)	0.010	1.917	1.82	(1.20, 2.76)	0.005	1.865
31–45	1.36	(1.08, 1.73)	0.038	1.693	1.44	(1.06, 1.97)	0.042	1.620
46–60	1.14	(0.96, 1.35)	0.145	1.524	1.01	(0.80, 1.27)	0.930	1.462
61 or older	1.00				1.00			
Sex								
Male	1.01	(0.88, 1.16)	0.870	1.083	0.91	(0.76, 1.09)	0.319	1.086
Female	1.00				1.00			
Enabling factor								
Income								
Q2	1.59	(1.35, 1.88)	<0.001	1.287	1.15	(0.91, 1.44)	0.238	1.236
Q3	1.56	(1.31, 1.86)	<0.001	1.313	1.30	(1.02, 1.65)	0.031	1.248
Q4	2.11	(1.70, 2.62)	<0.001	1.297	2.17	(1.66, 2.83)	<0.001	1.249
Q1	1.00				1.00			
Education								
Middle	0.92	(0.76, 1.11)	0.390	1.491	1.18	(0.93, 1.51)	0.181	1.513
High	0.85	(0.57, 1.27)	0.423	1.120	1.71	(1.09, 2.68)	0.019	1.147
Low	1.00				1.00			
Marital status								
Married	1.11	(0.87, 1.42)	0.388	2.740	1.60	(1.15, 2.22)	0.005	3.061
Divorced	1.19	(0.91, 1.57)	0.208	2.906	1.21	(0.83, 1.77)	0.329	3.194
Single	1.00				1.00			
Employment								
Yes	1.38	(1.18, 1.61)	<0.001	1.455	1.37	(1.11, 1.68)	0.003	1.428
No	1.00				1.00			
Place of residence								
Urban	1.17	(1.03, 1.34)	0.017	1.040	1.22	(1.02, 1.45)	0.046	1.037
Rural	1.00				1.00			
Need-for-care factor								
Chronic disease								
No	6.84	(5.93, 7.88)	<0.001	1.229	2.77	(2.30, 3.34)	<0.001	1.202
Yes	1.00				1.00			
Pearson’s GOF test								
Chi-square (DF)	1,749.290 (1,788)							
*p*-value	0.739							

AOR, adjusted odds ratio; CI, confidence interval; VIF, variance inflation factor; GOF, goodness-of-fit; DF, degrees of freedom.

Specifically, the MLR model of self-medication (vs. UCS) indicates that self-medication users tended to be young, have a high income, be employed, live in urban areas, and not have chronic diseases compared to UCS users. People aged 15–30 and 31–45 years were 1.53 and 1.36 times more likely to use self-medication than those aged 61 years or older. However, the use of self-medication was not different between people aged 46–60 and those aged 61 years or older.

In terms of income, people in Q2, Q3, and Q4 were 1.59, 1.56, and 2.11 times more likely to use self-medication than those in Q1. Employed people and those living in urban areas had a 1.38 and 1.17 times higher use of self-medication than unemployed and rural people, respectively. Lastly, people without a chronic disease were 6.84 times more likely to use self-medication than those with a chronic disease.

In addition, the MLR model of private providers (vs. UCS) reveals that private-provider users were more likely to be young, have a high income, be highly educated, married, and employed, and not have a chronic disease than UCS users. People aged 15–30 and 31–45 years had a 1.82 and 1.44 times higher use of private providers than those aged 61 years or older. However, people aged 46–60 years and those aged 61 years or older were not different in the use of private providers.

Regarding income, people in Q3 and Q4 were 1.30 and 2.17 times more likely to use private providers than those in Q1. However, the use of private providers was not different between people in Q2 and Q1. In terms of education and marital status, people who were highly educated and those who were married had a 1.71 and 1.60 times higher use of private providers than those who had a low education level and those who were single, respectively. Moreover, urban residents had a 1.22 times higher use of private providers than rural residents. Lastly, beneficiaries with a chronic disease were 2.77 times more likely to use private providers than those without a chronic disease.

In summary, consistent with the descriptive analysis results, the MLR results indicated that non-users of the UCS (both self-medication and private-provider users) generally had high socio-demographic status compared to UCS users. The common factors for the use of self-medication and private providers were a young age, high income, employment, urban residency, and the absence of a chronic disease.

### 3.3 Reasons for non-use of the UCS

The results regarding reasons for not using the UCS are revealed in Table 3. Among a total of 2,598 beneficiaries using private healthcare, 1,566 were missing values and 91 were unidentified values, such as “others” and “unknown.” After eliminating these missing and unidentified values, 941 beneficiaries were ultimately used for analysis.

**Table 3:** Reasons for non-use of the UCS.

Reasons for non-use of the UCS	Overall (*n* = 941; 100%)		Self-medication (*n* = 372; 39.5%)		Private provider (*n* = 569; 60.5%)	
	*n*	%	*n*	%	*n*	%
Availability barriers						
Limited service hours of designated provider	156	16.6	72	19.4	84	14.8
No designated provider in my place of residence	15	1.6	9	2.4	6	1.1
Limited healthcare benefits of the UCS	68	7.2	31	8.3	37	6.5
Subtotal	239	25.4	112	30.1	127	22.3
Accessibility barriers						
Long distance to designated provider	51	5.4	23	6.2	28	4.9
No transportation options to designated provider	3	0.3	2	0.5	1	0.2
Long queue in designated provider	507	53.9	194	52.2	313	55.0
Subtotal	561	59.6	219	58.9	342	60.1
Acceptability barriers						
Discrimination	1	0.1	1	0.3		
Unfriendly doctors or nurses	8	0.9	2	0.5	6	1.1
Subtotal	9	1.0	3	0.8	6	1.1
Quality barriers						
Unsureness of medicine quality	80	8.5	22	5.9	58	10.2
Inaccurate diagnosis and ineffective treatment	52	5.5	16	4.3	36	6.3
Subtotal	132	14.0	38	10.2	94	16.5
Total	941	100	372	100	569	100

Among the 941 non-users of the UCS, the most frequently reported reason for not using the UCS was accessibility barriers (59.6%), followed by availability (25.4%) and quality barriers (14%). Specifically, among the accessibility barriers, long queue in designated providers (53.9%) and long distance to designated providers (5.4%) were the main reasons. Among the availability barriers, the limited service hours of designated providers (16.6%) and limited healthcare benefits of the UCS (7.2%) were the main reasons. Among the quality barriers, unsureness about medicine quality (8.5%) and inaccurate diagnosis and ineffective treatment (5.5%) were the main reasons.

This pattern was consistent for both self-medication and private-provider users. However, an interesting difference was noticed between these two groups. Specifically, although the percentage of respondents who cited accessibility barriers was similar between self-medication (58.9%) and private-provider users (60.1%), the percentage of respondents who cited availability barriers was higher in self-medication users (30.1%) than in private-provider users (22.3%). Meanwhile, the percentage of respondents who cited quality barriers was higher in private-provider users (16.5%) than in self-medication users (10.2%).

In summary, the most common reason for not using the UCS was accessibility barriers (59.6%, e.g., long wait time), followed by availability (25.4%, e.g., limited service hours) and quality barriers (14%, e.g., unsureness of medicine quality), for both self-medication and private-provider users. Moreover, self-medication users tended to be concerned about availability issues, while private-provider users tended to be concerned about quality issues when using the UCS.

## 4 Discussion

Although the UCS has increased overall healthcare use by offering free healthcare since it began in 2002, a significant number of beneficiaries have continued to depend on private healthcare. This resultant low use of the UCS has been cited as a challenge to achieving the policy’s objective of universal access to healthcare. Thus, this study divided healthcare use into three patterns (self-medication, private providers, and UCS) and investigated the socio-demographic characteristics of non-users of the UCS and their reasons for non-use.

The results reveal that about 46.1% of UCS beneficiaries using healthcare services used them outside the UCS delivery system. Among them, about 33.8 and 12.3% used self-medication and private healthcare providers, respectively. The non-users generally had a higher socio-demographic status than the UCS users. Specifically, they were young, had a high income, were employed, lived in urban areas, or did not have a chronic disease. The most common reason for the non-use of the UCS was accessibility barriers (59.6%; e.g., long queues in public providers), followed by availability (25.4%; e.g., limited operating hours of public providers), and quality barriers (14%; e.g., unsureness about the quality of medicine given by public providers). Moreover, self-medication users tended to be concerned about availability barriers, while private-provider users tended to be concerned about quality barriers to using the UCS.

The results, especially those regarding the non-use rate of the UCS and associated socio-demographic patterns, were consistent with those from previous studies in Thailand (11–13). Moreover, this study explored reasons for the non-use of the UCS according to four components suggested by the WHO’s AAAQ framework (24, 25). In doing so, this study could assess specific barriers to the use of the UCS by patterns of healthcare use, which have been understudied in previous studies in Thailand.

The present results imply that access to healthcare is a multi-dimensional concept containing various sub-elements as indicated by the WHO’s AAAQ framework. Accordingly, universal access to healthcare cannot be accomplished by lowering financial barriers to healthcare use alone. Instead, it also relies on how multiple elements are considered and improved together.

Specifically, the UCS could be considered an insurance policy that increases financial accessibility (i.e., free healthcare in designated providers). This could increase healthcare use by low socio-demographic groups, who generally cannot afford healthcare, as shown in this study. However, the policy may not yet be able to increase other elements, such as availability and physical accessibility elements, which are related to healthcare resources and infrastructure. This may have caused the UCS to create situations that could not serve the demands of beneficiaries, especially those in high socio-economic groups (e.g., long queues in designated providers), and ultimately forced them to use private healthcare.

This discussion above is supported by several empirical studies in Thailand (28, 29). For instance, an observational study conducted by NaRanong and NaRanong found that after the UCS was introduced, public providers were greatly congested and understaffed, which led to issues for UCS beneficiaries, such as long queues and insufficient physician consultation times. The issues tended to push some beneficiaries who previously used public healthcare providers—especially those with severe illnesses, time constraints, or a high socio-demographic status—into the private healthcare sector (28).

Therefore, the government should make efforts to enhance the use of the UCS to accomplish the policy’s objective. This study recommends specific policy measures based on the results and discussions.

First, regarding accessibility barriers, particularly long queues, to public provider use, the workforce of the primary healthcare system should be strengthened. In Thailand, there are 77 provinces with 878 districts. Each province and district has one or two major provincial-and district-level hospitals, along with a number of sub-district-level healthcare centers. In 2016, there were 156 provincial-level hospitals, 780 district-level hospitals, and 9,579 sub-district-level healthcare centers (30, 31).

However, sub-district healthcare centers, which are a main component of the primary healthcare system, are staffed by two or three nurses and trained healthcare volunteers rather than physicians, except for some representative healthcare centers in each administrative area. Since these centers offer only basic healthcare services, beneficiaries demanding complex healthcare are generally referred to district-and provincial-level hospitals (31). This could have been the main cause of long queues for public providers. Thus, the government should strengthen the workforce of the primary healthcare system by increasing the number of representative centers and assigning more physicians to centers to alleviate the issue of long queues for designated providers.

Second, in terms of availability barriers, especially the restricted operating hours of designated providers (from 9:00 a.m. to 5:00 p.m.), previous studies have consistently reported that beneficiaries who have time constraints, such as employed people, generally depend on private healthcare (11–13, 20). To resolve this issue, the flexibility of the service hours of public providers needs to be increased by identifying people’s healthcare needs after operating hours. The UCS also requires cooperation with private providers to, for example, cover a certain portion of the after-service-hours healthcare offered by private providers.

Third, regarding quality barriers, a general perception that the quality of private healthcare (or private providers) is better than that of public healthcare (or public providers) is widespread in Thailand. The government should change the perception through diverse public relations activities, such as public campaigns and education programs.

Although we recommend specific policy measures based on the key results of the study, these recommendations should be short-term measures. In the long term, the government must continue its endeavors to address the current lack and geographical imbalance of healthcare resources and infrastructure in the public sectors and the low involvement of private healthcare providers in the UCS policy, as these factors are well-known to hamper the use of the UCS in Thailand (1, 2, 30, 31).

This study includes limitations that must be considered in future studies. First, the 2019 HWS asked respondents to select only one main place where they received healthcare services; thus, this study did not consider individuals who received healthcare services from different places at the same time (e.g., UCS and self-medication or UCS and private providers). Likewise, respondents were asked to select only one primary reason for not using the UCS. However, UCS beneficiaries may have encountered multiple barriers simultaneously. For instance, long queues and long distances may be direct barriers, while quality issues may be indirect barriers. Moreover, since a significant number of respondents did not answer or gave an undefined answer to the question, we had to use only limited samples to analyze reasons for the non-use of the UCS. Due to these issues, the study results may include over-or under-generalization issues. The National Statistical Office of Thailand must consider these issues for future HWSs. Addressing this limitation, especially the issue of missing or unidentified answers would allow future studies to explore reasons for the non-use of the UCS according to different socio-demographic groups so that policy measures can be modified accordingly.

Second, this study simplified the pattern of healthcare use into three categories (i.e., self-medication, private providers, and UCS). Such simplistic categorization might not accurately capture the policy impact. Specifically, regarding self-medication, we combined over-the-counter drugs and Thai traditional medicines. However, some Thai traditional medicines are covered by the UCS and are available in large provincial-and district-level hospitals (13). Additionally, there may be a possibility that some beneficiaries may have visited pharmacies to receive prescription medicines that are covered by the UCS.

Regarding private providers, some beneficiaries may have received healthcare services from UCS-contracted private providers, though these contracted providers cover only 5.7% of all UCS beneficiaries (13). However, the 2019 HWS did not provide related information in detail; accordingly, this study ultimately adopted the same categorization method employed in previous studies (11–13), which should also be considered by the National Statistical Office for future HWSs.

Third, the pattern of healthcare use relies generally on disease severity (32, 33). However, since the 2019 HWS did not include clinical factors, such as disease severity, this study did not control for them in the analysis. Particularly regarding self-medication, previous studies showed that the main reasons for using self-medication included mild symptoms, time-saving, convenient accessibility, and a scarcity of physicians (34–36). These results are partly consistent with our study’s results that UCS beneficiaries used self-medication primarily due to long queues and limited service hours of public providers. Nevertheless, future studies should consider adjusting for clinical factors including disease severity in analysis to make more precise evaluations of the policy’s effects on the pattern of healthcare use. Combining the HWS with other clinical data such as medical claims or hospital management data can help overcome this limitation.

Fourth, the study’s outcome variable (patterns of healthcare use) was measured by using self-reported data, with respondents directed to evaluate their healthcare needs by their perceptions. Thus, they should be aware of their healthcare needs and also be willing to report them. Such subjective measure unavoidably comprises recall bias and social desirability bias issues (37, 38); thus, this study’s results must be interpreted by considering this issue.

Lastly, the validity and reliability of the cross-sectional pattern of healthcare use found in this study should be examined in a longitudinal study. In addition, this study recommends that the government set the non-use of the UCS as a policy assessment indicator and establish a regular-basis monitoring system accordingly.

## 5 Conclusion

The results of the present study imply that healthcare access is a multi-dimensional concept containing diverse sub-elements, such as physical and financial accessibility of healthcare. Accordingly, universal access to healthcare cannot be readily accomplished by lowering financial barriers to healthcare use alone. Instead, it also relies on how multiple elements are considered and improved together.

In the case of the UCS, among the elements, the lack of healthcare resources and infrastructure in the public sectors was the most important issue in accomplishing its goal of universal access to healthcare. The UCS has increased financial accessibility for the use of the UCS (i.e., free healthcare from public providers). However, it probably has not yet increased healthcare resources and infrastructure facilitating the use of the UCS. This may have caused the UCS to create situations that could not serve the demands of beneficiaries, especially those in high socio-economic groups (e.g., long queues in public providers and limited service hours of public providers), and ultimately forced them to use private healthcare. Therefore, the government should continue its endeavors to invest in healthcare resources and infrastructure in the public sectors to increase the use of the UCS.

## Data Availability

Publicly available datasets were analyzed in this study. This data can be found: the 2019 dataset used in the present study is available from the authors upon reasonable request and with the permission of the National Statistical Office of Thailand. Requests to access the dataset should be directed to SCP, seungchun.pak@mahidol.ac.th.

## Appendix

**Table A1:** Complete results of multinomial logistic regression analysis.

Variables	Self-medication (vs. UCS)							Private provider (vs. UCS)						
	COR	95% CI	*p*-value	AOR	95% CI	*p*-value	VIF	COR	95% CI	*p*-value	AOR	95% CI	*p*-value	VIF
Predisposing factor														
Age														
15–30	3.95	(3.15, 4.94)	<0.001	1.53	(1.11, 2.11)	0.010	1.917	3.47	(2.57, 4.68)	<0.001	1.82	(1.20, 2.76)	0.005	1.865
31–45	3.30	(2.76, 3.94)	<0.001	1.36	(1.08, 1.73)	0.038	1.693	3.10	(2.44, 3.95)	<0.001	1.44	(1.06, 1.97)	0.042	1.620
46–60	1.99	(1.74, 2.28)	<0.001	1.14	(0.96, 1.35)	0.145	1.524	1.64	(1.34, 1.99)	<0.001	1.01	(0.80, 1.27)	0.930	1.462
61 or older	1.00			1.00				1.00			1.00			
Sex														
Male	1.15	(1.02, 1.29)	0.023	1.01	(0.88, 1.16)	0.870	1.083	1.05	(0.89, 1.24)	0.573	0.91	(0.76, 1.09)	0.319	1.086
Female	1.00			1.00				1.00			1.00			
Enabling factor														
Income														
Q2	1.71	(1.48, 1.98)	<0.001	1.59	(1.35, 1.88)	<0.001	1.287	1.30	(1.05, 1.62)	0.019	1.15	(0.91, 1.44)	0.238	1.236
Q3	1.92	(1.64, 2.24)	<0.001	1.56	(1.31, 1.86)	<0.001	1.313	1.67	(1.33, 2.09)	<0.001	1.30	(1.02, 1.65)	0.031	1.248
Q4	2.50	(2.08, 3.01)	<0.001	2.11	(1.70, 2.62)	<0.001	1.297	2.87	(2.25, 3.66)	<0.001	2.17	(1.66, 2.83)	<0.001	1.249
Q1	1.00			1.00				1.00			1.00			
Education														
Middle	2.00	(1.74, 2.31)	<0.001	0.92	(0.76, 1.11)	0.390	1.491	2.21	(1.82, 2.69)	<0.001	1.18	(0.93, 1.51)	0.181	1.513
High	1.93	(1.37, 2.73)	<0.001	0.85	(0.57, 1.27)	0.423	1.120	3.45	(2.32, 5.15)	<0.001	1.71	(1.09, 2.68)	0.019	1.147
Low	1.00			1.00				1.00			1.00			
Marital status														
Married	0.68	(0.57, 0.82)	<0.001	1.11	(0.87, 1.42)	0.388	2.740	0.90	(0.69, 1.17)	0.418	1.60	(1.15, 2.22)	0.005	3.061
Divorced	0.46	(0.38, 0.56)	<0.001	1.19	(0.91, 1.57)	0.208	2.906	0.48	(0.35, 0.65)	<0.001	1.21	(0.83, 1.77)	0.329	3.194
Single	1.00			1.00				1.00			1.00			
Employment														
Yes	2.27	(2.01, 2.55)	<0.001	1.38	(1.18, 1.61)	<0.001	1.455	2.02	(1.70, 2.40)	<0.001	1.37	(1.11, 1.68)	0.003	1.428
No	1.00			1.00				1.00			1.00			
Place of residence														
Urban	1.17	(1.04, 1.31)	0.008	1.17	(1.03, 1.34)	0.017	1.040	1.12	(0.95, 1.32)	0.186	1.22	(1.02, 1.45)	0.046	1.037
Rural	1.00			1.00				1.00			1.00			
Need-for-care factor														
Chronic disease														
No	7.81	(6.85, 8.90)	<0.001	6.84	(5.93, 7.88)	<0.001	1.229	3.47	(2.93, 4.12)	<0.001	2.77	(2.30, 3.34)	<0.001	1.202
Yes	1.00			1.00				1.00			1.00			
Pearson’s GOF test														
Chi-square (DF)	1,749.290 (1,788)													
*p*-value	0.739													

COR and AOR, crude and adjusted odds ratio; CI, confidence interval; VIF, variance inflation factor; GOF, goodness-of-fit; DF, degrees of freedom.
